# Cough Suppression during Flexible Bronchoscopy Using Transcutaneous Electric Acupoint Stimulation: A Randomized Controlled Study

**DOI:** 10.1155/2019/5650413

**Published:** 2019-11-19

**Authors:** Wei Zhang, Yi-Xiao Yang, Wei Yu, Si-Hua Qi

**Affiliations:** Department of Anesthesiology, The Fourth Affiliated Hospital of Harbin Medical University, Yiyuan Street 37#, Harbin 150001, Heilongjiang Province, China

## Abstract

*Background and Objectiv*e. Transcutaneous electric acupoint stimulation (TEAS) is recommended for its sedative and analgesic effects. We sought to evaluate the effect of TEAS on cough suppression during flexible bronchoscopy (FB) and explore the underlying mechanism. *Methods*. In this single-center, randomized, single-blind, parallel-controlled study, we randomized 100 patients scheduled for FB into two equal groups treated with or without TEAS (TEAS group and control group). Patients in the TEAS group received 30 min of *stimulation* at the Hegu (LI4), Neiguan (PC6), and Lieque (LU7) points before FB. The control group underwent the same procedure, but without stimulation. The primary outcome was the intraoperative cough score determined by the bronchoscopist. The secondary outcomes were patient-reported discomfort scores and other procedural parameters. *Results*. Compared with the controls, patients who received TEAS preconditioning had lower cough scores (*P*=0.0027) and requirement of lidocaine and fentanyl (*P* < 0.05) and significantly higher postprocedural plasma *β*-endorphin levels (*P*=0.0367). There were no intergroup differences in discomfort scores, midazolam dosage, rate of premature termination, oxygen requirement, sedation level, airway assistance, oxygen saturation, lowest oxygen saturation level, heart rate, plasma substance-P levels, and rate of complications after 24 h. The total procedure duration, time for passage of the bronchoscope through the vocal cords, and systolic and diastolic blood pressure levels were less in the TEAS group than in the control group (*P*=0.033, 0.039 and <0.05, respectively). *Conclusion*. The combination of midazolam and TEAS was superior to midazolam alone for cough suppression during FB, probably due to increased plasma *β*-endorphin levels. This trial is registered with ChiCTR1800016612 at chictr.org.cn/index.aspx.

## 1. Introduction

Flexible bronchoscopy (FB) is a widely performed procedure for the diagnosis and treatment of various bronchopulmonary disorders. During the procedure, cough is inevitable due to the intense stimulation of the airway surface. Many patients (25%) find that cough is the worst adverse effect of the procedure [1]. The current practice for FB is the combined administration of benzodiazepines and opiates for sedation and prevention of cough. However, the combined use of two sedatives raises the risk of respiratory depression [2]. Acupuncture is an important aspect of traditional Chinese Medicine (TCM), with a history spanning thousands of years. Various studies across China and elsewhere have provided evidence for the value of acupuncture in the treatment of cough and other respiratory disorders [3–5]. The effects of acupuncture have been reported to correlate with the level of endogenous opioid peptides [6] and substance-P [7, 8]. Transcutaneous electric acupoint stimulation (TEAS) is a method that combines the concepts of traditional acupuncture and modern electrophysiological therapy. Thus, in the present study, we aimed to investigate the effects of TEAS preconditioning on cough and discomfort in patients undergoing FB as well as the underlying mechanisms involved by assessing the levels of *β*-endorphin and substance-P.

## 2. Methods

### 2.1. Design

This was a randomized, single-blind, parallel-controlled, single-center prospective study trial designed to evaluate the effects of TEAS on cough and discomfort in patients undergoing FB.

### 2.2. Setting and Participants

We enrolled 100 patients aged 18–70 years who underwent FB at the Fourth Affiliated Hospital of Harbin Medical University between June 2018 and October 2018. In our pilot study comparing midazolam alone with midazolam and TEAS during FB, the mean (SD) cough scores were recorded to be 5.0 (±3.7) and 2.9 (±2.5), respectively. With a power of 0.8 and an acceptable type I error size of 0.05, the number of patients required in each arm was 44. To account for potential cases being lost to follow-up, the sample size was increased to 50 patients per arm.

#### 2.2.1. Inclusion Criteria

Patients who were scheduled for FB were included in this study if they met the following criteria: (1) bronchoscopy performed for the first time; (2) age between 18 and 70 years; (3) body mass index (BMI) between 18.5 and 35 kg/m^2^; (4) American Society of Anesthesiologists (ASA) physical classification system status of I, II, or III.

#### 2.2.2. Exclusion Criteria

Patients were excluded from this study if they (1) were undergoing emergency bronchoscopy; (2) were unable to provide informed consent; (3) had an allergy to any of the medicines used in this study; (4) underwent endobronchial ultrasound; (5) resting hypoxemia, oxygen saturation <90%; (6) had asthma or a forced expiratory volume in one second (FEV1) of <1 L; (7) were pregnant; and (8) had a baseline systolic pressure of >160 mmHg or <90 mmHg; baseline heart rate of >100 beats/min or <50 beats/min; or any other arrhythmia.

### 2.3. Ethical Considerations

This study was conducted in accordance with the Declaration of Helsinki, and the study protocol was approved by Medical Ethics Committee of the Fourth affiliated Hospital of Harbin Medical University-approval 2018-SCILLSC-01. This trial was registered with Chinese Clinical Trial Registry (trial number ChiCTR1800016612). Written informed consent was obtained from all study participants.

### 2.4. Randomization

In this study, the block-random method was used to carry out randomization. Patients were randomly assigned to the treatment group (TG) or the control group (CG) in a 1 : 1 distribution on the basis of random, computer-generated random numbers. The details of randomization were sealed in opaque envelopes and only viewed by the acupuncturist just before the start of the TEAS session.

### 2.5. Blinding

The acupuncturist who conducted the TEAS session did not participate in any of the subsequent procedures. Gel electrodes were applied on the same acupoints for all patients, who were blinded to their group allocation. Since none of the patients included in this study had received TEAS treatment previously, they were not aware of the subjective feelings produced by TEAS. Moreover, the patients were advised that irrespective of whether they did or did not experience any stimulation, TEAS would be effective. Therefore, the patients would be under the impression that they had received actual TEAS stimulation. After the TEAS session, the patients underwent FB by the standard procedure. The anesthesiologists, bronchoscopy physicians, and nurses who performed the FB were not aware of the group allocation of the patients.

### 2.6. Monitor and Oxygen Supply

Once the patient was positioned on the examination table in the procedure room for FB, the patient's vital signs, namely, oxygen saturation, heart rate, and noninvasive arterial pressure, were measured and monitored thereafter. Oxygen at a concentration of 4 L/min was supplied to all patients via a nasal cannula. The oxygen delivery was increased to 10 L/min in case of the oxygen saturation <90%. If oxygen saturation of ≥90% could not be maintained with oxygen supplementation, airway assistance maneuvers (chin lift, jaw thrust, nasopharyngeal tube insertion, face mask placement, and manual ventilation) were performed.

### 2.7. TEAS Protocol

**Figure 1:**
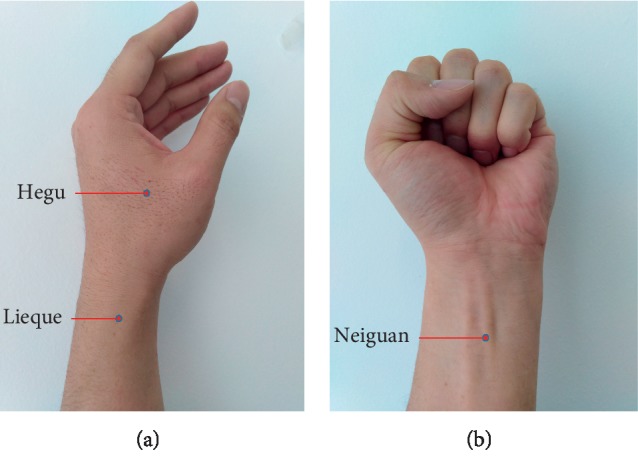
Location of acupoints. TEAS was applied to bilateral Hegu (LI4), Neiguan (PC6), and Lieque (LU7). LI4 is located on the back of the hand, between the 1st and 2nd metacarpal bones, and approximately at the middle of the second metacarpal bone on the radial side; PC6 is located on the palmar aspect of the forearm, 2 cuns above the transverse crease of the wrist between the flexor carpi radialis and palmaris longus tendons; and LU7 is located on the radial margin of the forearm, above the styloid process of the radius, 1.5 cuns above the transverse crease of the wrist between the brachioradialis muscle and the tendon of the abductor pollicis longus. Cun is unit of length of TCM. TCM considers the width of the interphalangeal joint of the thumb to be 1 cun of that of the patient. TEAS, transcutaneous electric acupoint stimulation; TCM, traditional Chinese medicine.

TEAS was bilaterally applied to the Hegu (LI4), Neiguan (PC6), and Lieque (LU7) points with a circular electrode of diameter of 0.5 cm by an experienced acupuncturist 30 min before FB. According to TCM, these acupoints were identified (Figure 1). Hegu and Neiguan form a circuit on the left side, while Hegu and Neiguan form a circuit on the right side; Lieque of both sides form a circuit. The acupoints were stimulated electrically using the Hwatuo electronic acupuncture treatment instrument (model no. SDZ-V, Suzhou Medical Appliances Ltd., Suzhou, China) at the same time. The stimulation parameters were set to a dense-disperse frequency of 2/10 Hz and an intensity of 6–9 mA. The waveform is an asymmetric bidirectional pulse wave. The pulse duration is sparse wave work for 5 seconds, followed by dense wave works 10 seconds. At the same time, the two-sided 3 pairs of acupoints were stimulated by the 2 Hz wave, and then, the two-sided 3 pairs of acupoints were stimulated by the 10 Hz wave. The sparse wave and the dense wave were alternately performed. The intensity was adjusted to cause the local muscles to twitch slightly, according to the maximum tolerance of each patient. In the control group, the same procedure of acupoint placement was followed, but the instrument was kept off, resulting in no electrical stimulation. All the electrodes and the stimulator were removed prior to the start of the FB; therefore, the bronchoscopist and nurse were blinded to whether the patient received true stimulation or sham stimulation.

### 2.8. Topical Anesthesia

Nasal anesthesia was achieved with 3 mL 2% lidocaine gel. The bronchoscopist sprayed 3 mL aliquots of 1% lidocaine solution over the oropharyngeal mucosa. With the patient in the supine position, a flexible bronchoscope (BF-1TQ180, Olympus, Tokyo, Japan) was introduced intranasally. Next, 3 mL aliquots of 1% lidocaine solution were sprayed onto the vocal cords, trachea, and bronchial tree through the FB using the “spray as you go” technique. Additional lidocaine was administered as per the discretion of the bronchoscopist.

### 2.9. Flexible Bronchoscopy

Sedation was induced after the TEAS treatment, with intravenous administration of midazolam (2 mg) and aliquots (1 mg) of midazolam titrated to achieve conscious sedation. The FB procedure was performed according to the clinical setting. Signs of pain or discomfort and persistent cough were considered as indicators of insufficient topical anesthesia; additional lidocaine was applied by the bronchoscopist. If lidocaine also failed to suppress the cough, intravenous fentanyl (0.5 mg) was administered, as per the bronchoscopist's discretion. The procedure was aborted, as per the bronchoscopist's judgment.

### 2.10. Collection of Data

Data were collected at five time points: T0, baseline; T1, just after TEAS treatment; T2, when the FB was introduced into the nasal cavity; T3, when the FB passed through the vocal cords; T4, after withdrawal of the bronchoscope; and T5, 2 h after transfer to the postanesthesia care unit (PACU).

The primary outcome was the cough score. The secondary outcomes were as follows: discomfort score, hemodynamic variables, respiratory variables, depth of sedation, dosage of local anesthesia and sedative, duration of the procedure, complications of the procedure, failure rate, and plasma levels of *β*-endorphin and substance-P.

The cough scores were assigned by the bronchoscopist at T4 using the visual analogue scale (VAS) by using a ruler about 10 cm in length with 10 scales on one side; 0 points indicate no cough, while 10 points represent the most severe cough. For all patients in the study, bronchoscopy and grading of the cough score were performed by the same endoscopist. Before the start of the study, the bronchoscopist used the VAS method to assess the degree of cough in 20 patients undergoing FB, in order to gain familiarity with the VAS scoring method. At T5, the patients were asked to grade the discomfort with the procedure using the VAS. Blood pressure, heart rate, and level of oxygen saturation were recorded at T0, T1, T2, T3, and T4. The depth of sedation was evaluated at T1, T2, T3, and T4 using the modified observer's assessment of alertness/sedation (MOAA/S) scale. Duration from T2 to T3 and from T2 to T4 was recorded. The complications were evaluated 24 h after the procedure. For the measurement of the levels of *β*-endorphin and substance-P, 2 mL of venous blood was drawn from the cubital vein at T0, T1, and T4 and transferred into precooled tubes containing EDTA and trasylol. The samples were immediately centrifuged at 3000*g* for 10 min, and the isolated plasma was stored at −80°C until analysis. The levels of *β*-endorphin and substance-P were measured with commercially available enzyme-linked immunosorbent assay (ELISA) kits (Shanghai Enzyme-linked Biotechnology Co., Ltd).

### 2.11. Data Analyses

In our preliminary study, the cough score in the control group was 5.0 ± 3.7; accordingly, a reduction of the score from 5.0 to 3.0 was considered to indicate a clinically significant improvement by bronchoscopists at our hospital. To achieve a significant level of <0.05 with a power of 0.8, 44 patients were required in each study arm. Accounting for a 10% drop-out rate, we determined that the sample size required for each arm was 50.

All statistical analyses were performed using SPSS 22.0 (SPSS, Inc., Chicago, IL, USA). Intergroup differences in the dichotomous variables were evaluated using the chi-squared test or Fisher's exact test, as appropriate; data are presented as *n* (%). Normally distributed parameters were analyzed using Student's *t*-test for equality of means, with data presented as mean ± standard deviation (SD). Continuous, non-normally distributed parameters were evaluated using the Wilcoxon test, as appropriate, and the data are presented as median (interquartile range) [9]. The blood pressure, heart rate, and pulse oxygen saturation changes over time were analyzed using ANOVA. A *P* value of <0.05 was considered significant.

## 3. Results

**Figure 2:**
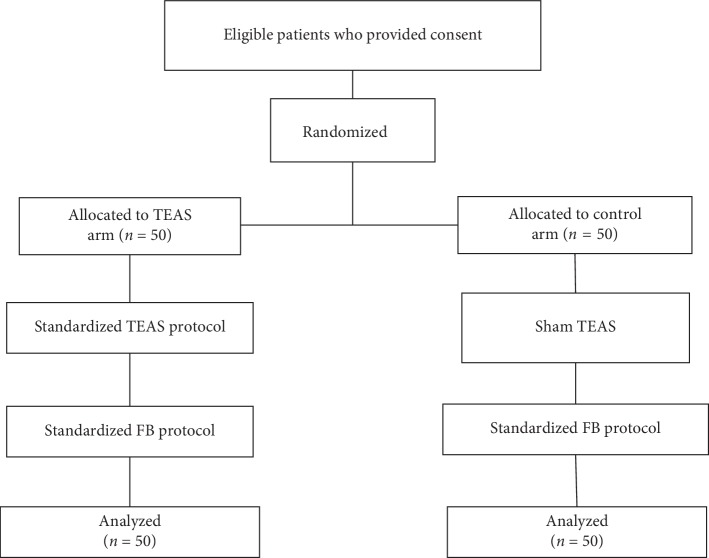
Flow diagram. One hundred patients were randomized, and all patients completed the study. TEAS, transcutaneous electric acupoint stimulation; FB, flexible bronchoscopy.

All the 100 patients completed the study, and all the data were analyzed (Figure 2). The basic demographic and clinical characteristics of all enrolled patients are presented in Table 1. The two groups did not show any significant difference in terms of sex distribution, age, height, BMI, ASA class, Mallampati score, smoking, indications for FB and the diagnostic process, and current medication, except for the frequency of infection.

Table 2 compares the data of the procedure-related parameters in the two groups. The data indicate that compared with the control group, those in the TEAS group had lower cough scores (*P*=0.0027), lower lidocaine requirement (*P*=0.0001), and lower requirement of fentanyl (*P*=0.0166). Furthermore, the total duration of T2∼T4 and T2∼T3were shorter in the TEAS group than in the control group (*P*=0.033 and 0.039, respectively). The two groups did not differ in terms of discomfort scores, dosage of midazolam, oxygen flow, lowest oxygen saturation, and depth of sedation. In one case, each in both the groups, the procedure could not be completed because of uncontrollable cough.

Table 3 compares the occurrence of adverse events in the two groups. The results showed that there was no significant difference between the two groups in the adverse events.

**Figure 3:**
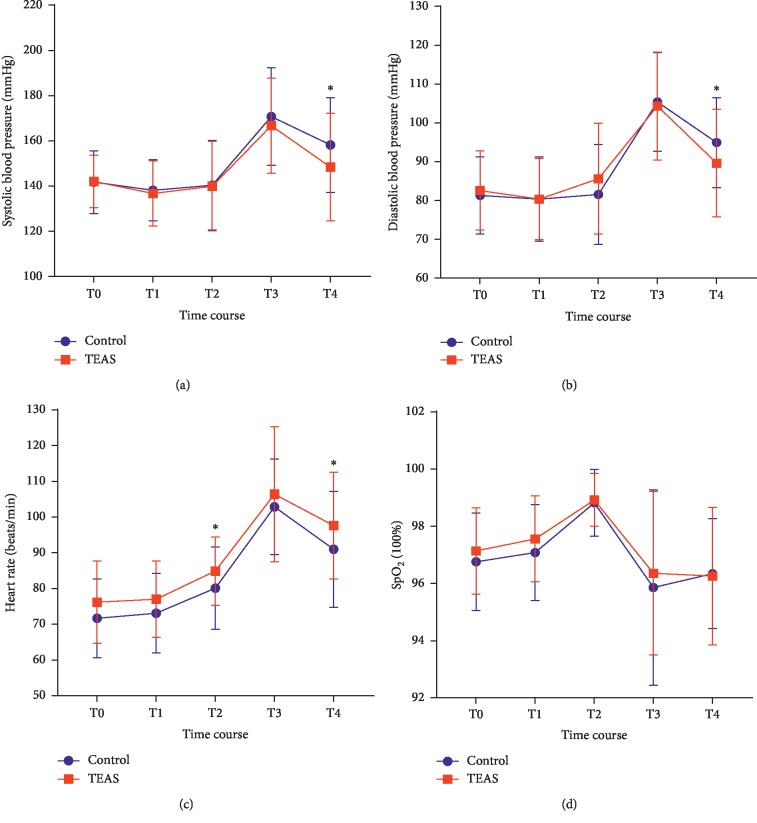
Changes in systolic blood pressure, diastolic blood pressure, heart rate, and oxygen saturation. Results are presented as mean (±SD). SpO_2,_ oxygen saturation; TEAS group, transcutaneous electric acupoint stimulation group; T0, baseline; T1, just after TEAS treatment; T2, insertion of the bronchoscope; T3, passage of the bronchoscope through the vocal cords; T4, after withdrawal of the bronchoscope. ^*∗*^*P* < 0.05.

Repeated-measures ANOVA showed that the systolic blood pressure as well as the diastolic blood pressure levels overall and at T4 were lower in the TEAS group than those in the control group (*P*=0.0442) (Figures 3(a) and 3(b)).

Similarly, the heart rate in the TEAS group was greater than that in the control group at T2 and T4 (Figure 3(c)). However, the intergroup difference in the heart rate during the FB procedure was not found to be significant (ANOVA; *P*=0.8045).

The oxygen saturation levels in the two groups were comparable (Figure 3(d)).

**Figure 4:**
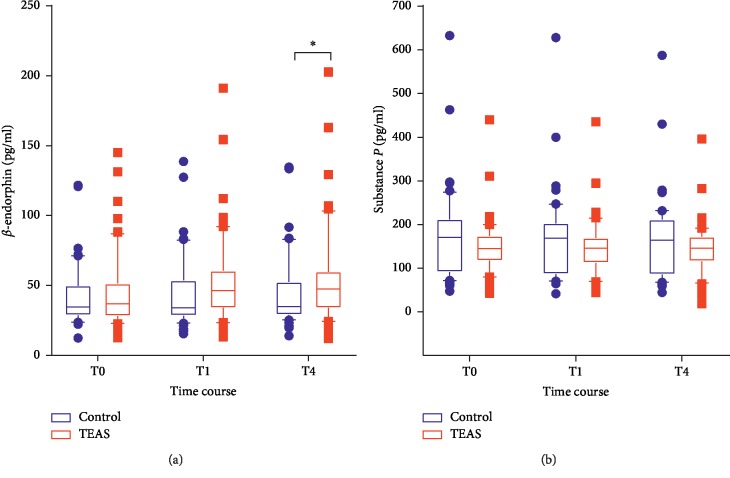
Plasma levels of *β*-endorphin and substance-P. Results are presented as median (IQR). TEAS group, transcutaneous electric acupoint stimulation group; T0, baseline; T1, just after TEAS treatment; T4, after withdrawal of the bronchoscope. ^*∗*^*P* < 0.05.

The data in Figure 4(a) show that the plasma level of *β*-endorphin at T1 was higher in the TEAS group than in the control group, but the difference was not significant (*P*=0.0657). At T4, the plasma level of *β*-endorphin in the TEAS group was significantly higher than that in the control group (*P*=0.0367). There was no intergroup difference in the plasma level of substance-P between the two groups (Figure 4(b)).

## 4. Discussion

**Figure 5:**
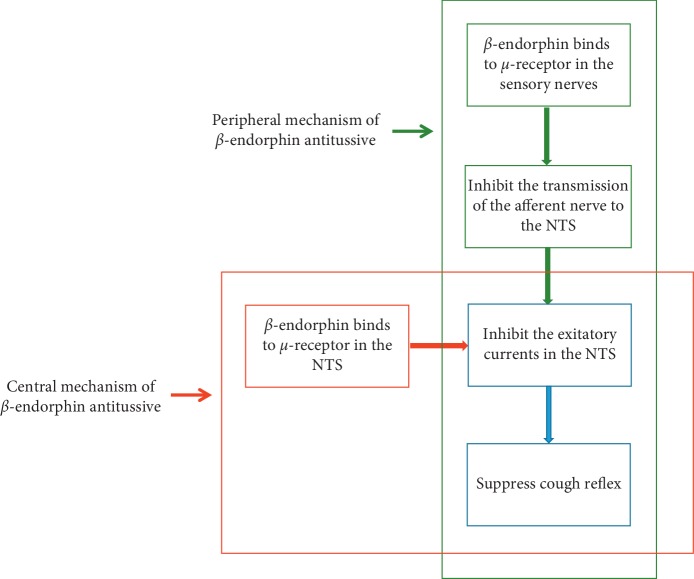
Mechanism of *β*-endorphin antitussive. NTS, nuclei tractus solitarii.

The major findings of this study on cough suppression in FB are as follows: compared with the use of midazolam alone, the combined application of TEAS preconditioning and midazolam (1) provided better cough suppression, (2) reduced the required dosage of lidocaine and fentanyl, (3) decreased the duration of the procedure, (4) stablized the blood pressure, and (5) increased the release of *β*-endorphin. We speculate that *β*-endorphin can suppress cough through central and peripheral pathways (Figure 5).

A study by Qi et al. showed that TEAS is safe and effective for FB examination; their results showed that TEAS could reduce the required dosage of sedatives and decrease the waking time [10]. However, theirs was an open-label study, which may lead to some bias in the results of the study, and patient-centered parameters such as the degree of cough and discomfort of the patients were not assessed. Furthermore, Qi et al. did not explore the mechanism by which TEAS exerts these effects.

To the best of our knowledge, this is the first randomized, single-blind, placebo-controlled study on the effect of combination of TEAS preconditioning and midazolam in FB. The discomfort experienced during FB includes cough, nausea, pain, and dyspnea [11]. Cough is an inevitable adverse effect associated with FB; in fact, 25% of patients undergoing FB consider cough as the worst adverse effect of bronchoscopy [1]. Severe coughing during transbronchial biopsy may increase the risk of pneumothorax [12]. Cough suppression is very important for the quality of the bronchoscopy and facilitates the bronchoscopic visualization and biopsy [13]. The antitussive effect of TEAS preconditioning in flexible bronchoscopy has good clinical significance. The sensitivity of cough is influenced by many factors, including respiratory tract infection [14]. Respiratory tract infections increase cough sensitivity and make patients more prone to cough reflexes to stimulation. The results showed that frequency of respiratory tract infection in the TEAS group was higher than that in the control group. If other factors are not taken into account, we think that the cough sensitivity of patients in TEAS group may be higher, but the results of the study showed that the cough score of patients in TEAS group is lower than that in the control group. Therefore, we believe that the antitussive effect of TEAS overrides the cough sensation associated with the infection.

In the current study, the cough score for all subjects was rated by the same bronchoscopist, by using the VAS. There are many ways to assess cough, and VAS is one of the commonly used methods [15]. Since cough affects the operation of the FB [13], it is more appropriate for the bronchoscopist to assess the extent of cough.

The results of this study showed that the median discomfort scores of the two groups were 2 and 1, respectively. Although the difference was not statistically significant, it could be inferred that TEAS tended to reduce the discomfort of patients. Previous studies have demonstrated that TEAS is effective in inducing analgesia [16] and in reducing the incidence of postoperative nausea [17], which may explain the patient's self-reported discomfort scores. All patients in this study received midazolam, and the discomfort scores of both arms might be induced to low by the amnesic effect of midazolam.

The results of the current study correlate with those of a previous study [18], which showed that TEAS increases the release of *β*-endorphin. *β*-endorphin has been shown to activate the *μ*-opioid receptor and to have a therapeutic effect [19, 20]. The study by Kamei et al. suggested that the antitussive effect of *β*-endorphin was mediated by the activation of the *μ*-receptor in nucleus tractus solitarii (NTS) [21]. In our study, we observed an increase in the plasma *β*-endorphin levels and not in the NTS; however, the study by Clement-Jones et al. showed that acupuncture increased the level of opioid peptide in the cerebrospinal fluid [22]. These results suggested that the level of *β*-endorphin in the central nervous system may be correlated with the level of *β*-endorphin in the peripheral blood.

Additionally, Callaway et al. showed that the use of aerosolized *μ*-receptor agonist resulted in significant cough inhibition, which implies that peripheral *μ*-receptor has antitussive properties [23]. Therefore, we believe that the TEAS-induced increase in the release of *β*-endorphin activates the peripheral *μ*-receptor in the airway, thereby suppressing cough.

We measured the plasma level of substance-P because it has been shown to be important for cough by inducing vasodilation, mucous secretion, edema, and bronchoconstriction [24, 25]. Within the brainstem, substance-P sensitizes the airway reflex driven by mechanically sensitive afferent input [26]. However, the data in our study did not show any effect of TEAS on the plasma levels of substance-P. Thus, we can only suggest that the antitussive effect of TEAS might not be mediated by changes in the plasma substance-P level.

Our data suggested that TEAS preconditioning decreased the requirement of lidocaine and fentanyl. This reduced requirement of lidocaine and fentanyl may be explained by the lower cough score in the TEAS group. It is reasonable to assume that minimal application of the topical anesthetic ensures the safety of the patients. Fentanyl is recommended as the preferred opioid agent [27]. The combined use of midazolam and fentanyl has a synergistic effect on patient tolerance and antitussive properties [27]. However, the combination of midazolam and fentanyl may increase the risk of hypoxemia and apnea [28]. Additionally, unlike the case with other countries, bronchoscopists and nurses in China generally have no experience with fentanyl and its opioid derivatives; therefore, we did not apply fentanyl, unless administration of additional lidocaine failed to effectively suppress the cough.

The dose of midazolam administered and the depth of sedation were similar in both the study arms, implying that TEAS preconditioning does not have sedative action. Liu et al. have shown that TEAS enhances the sedative effect of propofol administered at low concentration [29]. The discrepancy between our results and those of Liu et al. may be attributed to the differences in the acupoints, stimulus, and evaluation method of the two studies.

We compared the duration of treatment in both study arms. The results indicated that TEAS preconditioning was associated with a shorter duration of the entire procedure (T2∼T4) as well as reduced time to insertion of the scope (T2∼T3). The antitussive effect of TEAS may contribute to the shorter duration of the procedure because cough control has been shown to facilitate FB [13]. However, we acknowledge that there are other factors that influence the duration of the FB procedure, such as complexity of the procedure. We think that the intergroup difference in the duration was so small that it had no clinical significance. Therefore, we cannot conclude that TEAS can reduce the duration of FB.

The respiratory parameters in the two groups were comparable. This suggests that TEAS preconditioning had no correlation with respiration-related parameters. The circulatory parameters indicated that TEAS preconditioning prevented the elevation of blood pressure during FB. This is consistent with the findings of studies that show that electroacupuncture has a suppressive effect on cardiovascular sympathetic reactions [30]. The blood pressure suppression may also be attributed to the antitussive effect and analgesic effect of TEAS.

The adverse events in the two groups were comparable, and none of the patients in either study arm developed any complication during the first 24 h after treatment. This finding is similar to those reported from a multicenter study [31], which showed the low incidence of early (initial 24 h) complications after FB. Moreover, this result could also be attributed to the less complex nature of the procedures in the current study.

Being naïve to the treatment may not be sufficient to blind participants. Although efforts are made in this study to keep patients unaware of their group allocation, during the intervention, the patients were awake, and they would experience electric stimulation or nothing. This may have had some influence on the results of this study.

The analgesic effects of electroacupuncture lasted 2 to 3 hours [6], so the authors speculate that it may also be beneficial to use TEAS in FB for longer procedures. Moreover, it has been suggested that TEAS during supratentorial craniotomy can significantly decrease intraoperative sufentanil requirements and improve postoperative outcome [32]; therefore, we speculate that TEAS during longer FB procedures would be effective in cough suppression.

The FB procedure applied in this study is relatively simple and short. Further investigation is necessary to determine whether the findings of this study can be extended to complex FB procedures such as transbronchial biopsy or endobronchial ultrasound.

## 5. Limitations

Our study does have a few limitations. First, the sample size was small, which implies that the results cannot be easily extrapolated to other populations. Second, we did not include a study arm with nonacupoint TEAS; therefore, we were unable to prove the acupoint specificity of TEAS. Third, among the parameters assessed in this study, we included only two plasma biomarkers. In our future studies, we aim to include more parameters to explain the mechanism of action of TEAS. Fourth, we did not assess the blinding of the experimenters or participants. Blinding assessment would be employed in future projects.

## 6. Conclusion

In conclusion, our findings revealed that the combination of TEAS preconditioning and midazolam is superior to midazolam alone in suppressing cough during FB, while also stabilizing blood pressure and reducing the required dosage of lidocaine and fentanyl. TEAS produced an improvement in FB via an increase in the plasma *β*-endorphin levels. On the basis of our findings, we believe that a combination of TEAS preconditioning and midazolam may be a cost-saving, noninvasive, nonpharmacologic, and alternative regimen for patients undergoing FB.

## Figures and Tables

**Table 1:** Baseline characteristics of 100 consecutive patients undergoing flexible bronchoscopy.

Characteristics	C group (*n* = 50)	T group (*n* = 50)	*P* value
Age (y)	54.00 (±11.47)	57.60 (±7.82)	0.0702

Male, *n*	27 (54.00%)	24 (48.00%)	0.5484

Weight (kg)	68.02 (±9.35)	67.08 (±9.50)	0.6191

Height (cm)	166.88 (±6.20)	166.94 (6.89)	0.9636

BMI (kg/m^2^)	24.37 (±2.69)	24.05 (±2.96)	0.5683

ASA class			
I	24 (48.00%)	21 (42.00%)	0.7190
II	24 (48.00%)	29 (58.00%)	
III	2 (4.00%)	0 (0.00%)	

Mallampati score			
I	18 (36.00%)	26 (52.00%)	0.0711
II	28 (56.00%)	23 (46.00%)	
III	4 (8.00%)	1 (2.00%)	

Current smoker	14 (28.00%)	13 (26.00%)	0.9708
Ex-smoker	14 (28.00%)	14 (28.00%)	
Nonsmoker	22 (44.00%)	23 (46.00%)	

Indication for bronchoscopy			
Infection	9 (18.00%)	20 (40.00%)	0.0153
Chronic cough	11 (22.00%)	5 (10.00%)	0.1017
Hemoptysis	3 (6.00%)	7 (14.00%)	0.1824
Suspicion of malignancy	19 (38.00%)	16 (32.00%)	0.5294
Cancer follow-up	4 (8.00%)	1 (2.00%)	0.1687
Chest pain	3 (6.00%)	1 (2.00%)	0.3074
Atelectasis	1 (2.00%)	0 (0.00%)	1.0000

Diagnostic procedures			
Inspection only	10 (20.00%)	16 (32.00%)	0.1713
Bronchial washing	26 (52.00%)	22 (44.00%)	0.4233
Bronchial brushing	1 (2.00%)	1 (2.00%)	1.0000
Endobronchial biopsy	13 (26.00%)	9 (18.00%)	0.3342
Combined procedures not including inspection	0 (0.00%)	2 (4.00%)	0.1531

Current medication			
Antitussive	3 (6%)	6 (12%)	0.4846
ACEI	1 (2%)	3 (6%)	0.6098

Data are presented as mean ± SD or *n* (%), unless otherwise stated. TEAS group: transcutaneous electric acupoint stimulation; BMI: body mass index; ASA: American Society of Anesthesiologists.

**Table 2:** Outcome parameters in the control group and TEAS group.

Characteristics	C group (*n* = 50)	T group (*n* = 50)	*P* value
Physician cough score; VAS	6.00 (3.00)	4.50 (2.00)	0.0027

Patient discomfort score; VAS	2.00 (2.00)	1.00 (1.00)	0.0747

Midazolam dose (mg)	3.00 (1.00)	3.00 (1.00)	0.2124

Lidocaine dose (mg)	240.00 (60.00)	210.00 (30.00)	0.0001

Requirement of fentanyl, *n* (%)	13 (26.00%)	4 (8.00%)	0.0166

Oxygen requirement, *n* (%)			
4 L/min	35 (70.00%)	41 (82.00%)	0.1601
10 L/min	15 (30.00%)	9 (18.00%)	

Lowest SpO_2_ (%)	92.12 (±4.80)	93.34 (±3.55)	0.1522

Duration of the bronchoscopy, min			
T2∼T3	2.34 ± 1.92	1.64 ± 1.05	0.039
T2∼T4	8.30 ± 4.32	6.76 ± 2.92	0.033

MOAA/S score			
T2	3.62 ± 0.49	3.74 ± 0.44	0.243
T3	3.90 ± 0.30	3.88 ± 0.33	0.766
T4	4.00 ± 0.00	3.94 ± 0.24	0.083

Data are presented as mean ± SD, median (interquartile range), or *n* (%). TEAS group, transcutaneous electric acupoint stimulation; VAS, visual analogue scale; MOAA/S, modified observer's assessment of alertness/sedation; T2, time point when bronchoscope was inserted nasally; T3, time point at which the bronchoscope passed the vocal cord; T4, time point just after the procedure was completed.

**Table 3:** Adverse events in the control group and TEAS group.

Characteristics	C group (*n* = 50)	T group (*n* = 50)	*P* value
SBP ≥180 mmHg, *n* (%)	28 (56.00%)	20 (40.00%)	0.1093

HR ≥100 beats min^−1^	31 (62.00%)	35 (72.00%)	0.3984

SpO_2_ ≤90%, *n* (%)	3 (6.00%)	1 (2.00%)	0.3074

Premature termination, *n* (%)	1 (2.00%)	1 (2.00%)	1.0000

Airway assistance, *n* (%)	0	0	1.0000

Complication after 24 h, *n* (%)			
Bleeding	0	0	1.0000
Pneumothorax	0	0	1.0000
Transfer to ICU	0	0	1.0000
Death	0	0	1.0000
Other complication	0	0	1.0000

Data are presented as *n* (%). TEAS group, transcutaneous electric acupoint stimulation; SBP, systolic blood pressure; HR, heart rates; SpO_2_, oxygen saturation; ICU, intensive care unit.

## Data Availability

The data that support the findings of this study are included within the supplementary information file.

## Abbreviations

TEAS: Transcutaneous electric acupoint stimulation
FB: Flexible bronchoscopy
TCM: Traditional Chinese medicine
BMI: Body mass index
ASA: American Society of Anesthesiologists
FEV1: Forced expiratory volume in one second
TG: Treatment group
CG: Control group
VAS: Visual analogue scale
MOAA/S: Modified observer's assessment of alertness/sedation
ELISA: Enzyme-linked immunosorbent assay
NTS: Nucleus tractus solitarii
PACU: Postanesthesia care unit.

## Supplementary Materials

Supplementary Materials 1. Column A is the order in which the patient is enrolled. 2. Column B: Arm: C, control arm; T, treatment arm. 3. Column C: gender. 4. Column D: age (year). 5. Column E: height (cm). 6. Column F: weight (Kg). 7. Column G: BMI (kg/m2): BMI, body mass index. 8. Column H: ASA class: ASA, American Society of Anesthesiologists. 9. Column I: Mallampati score. 10. Column J- smoker. 11. Column K- current medication. 12. Column L: SpO2 T0 (%): SpO2, oxygen saturation; T0, baseline. 13. Column M: SpO2 T1 (%): T1, just after TEAS treatment. 14. Column N: SpO2 T2 (%): T2, insertion of the bronchoscope. 15. Column O: SpO2 T3 (%): T3, passage of the bronchoscope through the vocal cords. 16. Column P: SpO2 T4 (%): T4, after withdrawal of the bronchoscope. 17. Column Q: SBP T0 (mmHg): SBP, systolic blood pressure. 18. Column R: SBP T1 (mmHg). 19. Column S: SBP T2 (mmHg). 20. Column T: SBP T3 (mmHg). 21. Column U: SBP T4 (mmHg). 22. Column V: DBP T0 (mmHg): DBP, diastolic blood pressure. 23. Column W: DBP T1 (mmHg). 24. Column X: DBP T2 (mmHg). 25. Column Y: DBP T3 (mmHg). 26. Column Z: DBP T4 (mmHg). 27. Column AA: HR T0 (beats/min): HR, heart rates. 28. Column AB: HR T1 (beats/min). 29. Column AC: HR T2 (beats/min). 30. Column AD: HR T3 (beats/min). 31. Column AE: HR T4 (beats/min). 32. Column AF: MOAA/S T2: MOAA/S, modified observer's assessment of alertness/sedation. 33. Column AG: MOAA/S T3. 34. Column AH: MOAA/S T4. 35. Column AI: duration T2–T4 (min). 36. Column AJ: duration T2–T3 (min). 37. Column AK: lowest SpO2 (%). 38. Column AL: O2 requirement (L/min). 39. Column AM: midazolam (mg): midazolam dose. 40. Column AN: lidocaine (mg): lidocaine dose. 41. Column AO: fentanil (Yes/No): use of fentanyl. 42. Column AP: premature termination (Yes/No): early termination for any reason. 43. Column AQ: indication: indication of bronchoscopy. 44. Column AR: diagnostic procedures. 45. Column AS: cough score (VAS): VAS, visual analogue scale. 46. Column AT: discomfort score (VAS). 47. Column AU: SP T0 (pg/mL): SP, substance-P. 48. Column AV: SP T1 (pg/mL). 49. Column AW: SP T4 (pg/mL). 50. Column AX: *β*-endorphin T0 (pg/mL). 51. Column AY: *β*-endorphin T1 (pg/mL). 52. Column AZ: *β*-endorphin T4 (pg/mL).
